# Genetic resistance - an alternative for controlling PRRS?

**DOI:** 10.1186/s40813-016-0045-y

**Published:** 2016-11-16

**Authors:** Gerald Reiner

**Affiliations:** Department of Veterinary Clinical Sciences, Swine Clinic, Justus-Liebig-University, Frankfurter Strasse 112, 35392 Giessen, Germany

**Keywords:** PRRS, Disease resistance, Gene editing, *CD163*

## Abstract

PRRS is one of the most challenging diseases for world-wide pig production. Attempts for a sustainable control of this scourge by vaccination have not yet fully satisfied. With an increasing knowledge and methodology in disease resistance, a new world-wide endeavour has been started to support the combat of animal diseases, based on the existence of valuable gene variants with regard to any host-pathogen interaction. Several groups have produced a wealth of evidence for natural variability in resistance/susceptibility to PRRS in our commercial breeding lines. However, up to now, exploiting existing variation has failed because of the difficulty to detect the carriers of favourable and unfavourable alleles, especially with regard to such complex polygenic traits like resistance to PRRS. New hope comes from new genomic tools like next generation sequencing which have become extremely fast and low priced. Thus, research is booming world-wide and the jigsaw puzzle is filling up – slowly but steadily. On the other hand, knowledge from virological and biomedical basic research has opened the way for an “intervening way”, i.e. the modification of identified key genes that occupy key positions in PRRS pathogenesis, like *CD163. CD163* was identified as the striking receptor in PRRSV entry and its knockout from the genome by gene editing has led to the production of pigs that were completely resistant to PRRSV – a milestone in modern pig breeding. However, at this early step, concerns remain about the acceptance of societies for gene edited products and regulation still awaits upgrading to the new technology. Further questions arise with regard to upcoming patents from an ethical and legal point of view. Eventually, the importance of *CD163* for homeostasis, defence and immunity demands for more insight before its complete or partial silencing can be answered. Whatever path will be followed, even a partial abolishment of PRRSV replication will lead to a significant improvement of the disastrous herd situation, with a significant impact on welfare, performance, antimicrobial consumption and consumer protection. Genetics will be part of a future solution.

## Background

The production of PRRS resistant pigs by gene editing has produced a milestone in pig breeding and a big hope for a sustainable combat of an important disease. However, much remains to be done to reach the demands of practical breeding. While gene editing methods try to modify genes that play important roles in the pathogenesis of a disease, functional genome analysis and association studies intend to detect and exploit naturally existing genetic variation in such genes. Both approaches are in process at a world-wide endeaver to improve resistance of pigs to infectious diseases. The aim of this review is to provide insight into status and potential of these applications with regrd to PRRS, with a glimpse on future and existing concerns. Conclusions are based on the existing literature.

## Genetic resistance as a first choice of prophylaxis

Breeding for disease-resistant pigs might be the *ultima ratio* in combatting infectious diseases. Regardless of whether pigs would be resistant *sensu stricto*, (i.e., the absolute prevention of an infection, or just tolerating the infection) minimal amplification and shedding of the pathogen and minimal effects on health and performance could be achieved. Thus, the infectious pressure in and between herds could be efficiently reduced, followed by diminished disease incidence, improved performance and product quality, reduced antibiotic treatment, improved consumer protection and increased animal welfare [90].

## Genetic resistance in practical breeding

Disease-resistant breeds, populations or animals are of considerable importance to livestock. Prime examples are resistance to coccidiosis and Marek’s disease in fowl (e.g. [20]), to trypanosomiasis [77] and ticks [81] in cattle at tropical sites, to mastitis in dairy cattle (e.g. [41]), and to gastro-intestinal nematodes in sheep [109]. In pigs, however, examples of genetic resistance in commercial breeding programmes are sparse. Two examples are resistance to fimbriated F18 [121] and F4 [49] *Escherichia coli*. They represent rare cases of single-gene controlled genetic resistance. F18 fimbriated E.coli cause post-weaning diarrhoea and oedema disease [73] and resistance is realised by a receptor variant that does not bind any type of E.coli F18 fimbriae. Similarly, the right F4 receptor variant gives resistance to neonatal diarrhoea caused by most of F4-fimbriated E.coli. Other examples include the breeding for improved immune responsiveness, i.e. a higher general reactivity in humoral and cellular immunity in pigs (e.g. [129]). In spite of the currently limited commercial applications in swine, a wide range of genetic variation has been observed in genetic resistance to different bacterial, viral and parasitic diseases. A comprehensive search for disease resistance might identify differences in susceptibility/resistance in any host-species with regard to any relevant pathogen [90]. However, most of this genetic variation cannot be used in practical breeding, because of the difficulty in recognising favourable and unfavourable gene variants within the breeders. Their identification is impeded by highly variable and influential farm-specific environmental effects (e.g., pathogen load, immunity, housing, feeding and management conditions), the polygenic inheritance mode of most resistance traits, the limited availability of animal models and limited detailed knowledge of pathogenesis for most porcine diseases.

## Improving genetic disease resistance

While classical breeding is, thus generally inappropriate for efficient improvement of genetic resistance, evolved knowledge of the porcine genome combined with new tools and technologies – developed in the context of genome projects – have created new opportunities to dissect the genetic control of complex traits, including host responses to infection [4]. Alternatively to classical breeding, responsible gene variants can be identified via experiments in selected populations that vary significantly in resistance/susceptibility, under standardised environmental conditions, including time point of challenge and quantity of the pathogen. Once the responsible gene variants are identified, causal variants in experimental populations need to be validated in commercial farms to confirm segregation and association, prior to application in selection. Then, breeders can be selected via marker-assisted and genomic selection [74]. Provided there is societal consent, desirable gene variants can even be introduced into breeding populations via genetic engineering (e.g., [84]). In addition, understanding the molecular basis of genetic resistance will help improving the knowledge of the underlying mechanisms of disease and disease resistance, thus promoting new and enhanced developments in diagnostics, therapy and prophylaxis.

## Examples on the way

We have seen there are limited examples of applicable gene variants already in the field to improve genetic resistance in swine [49, 121]. However, the search for significant and applicable gene variants has developed into an ever-expanding and successful branch of clinical research, including viral (Pseudorabiesvirus [88]; Influenza A (e.g. [143]); bacterial (*Haemophilus parasuis* [131]; *Actinobacillus pleuropneumoniae* [93, 94]; *Mycoplasma hyopneumoniae* (e.g., [108]); *Streptococcus suis* [136]) and parasitic diseases (*Sarcocystis* [89]; *Ascaris suum* [100, 101]). More than 2,500 quantitative trait loci (QTL) have been published for health parameters in the pig, among them 400 for resistance/susceptibility against a broad range of pathogens (http://www.animalgenome.org/QTLdb/; current status: October 2016). QTL are gene loci which participate in the control of quantitative distributed traits such as milk yield, growth performance and disease resistance. The most remarkable results have been seen in resistance to PRRSV.

## Porcine Reproductive and Respiratory Syndrome (PRRS)

PRRS is one of the most devastating diseases in swine, worldwide (for overview see [147]). The disease causes respiratory and reproduction failures. Losses for the US pig industry were estimated at over $ 650 million annually, excluding costs for diagnosis, vaccination, treatment and biosecurity [44]. PRRSV is a single stranded RNA virus from the *Arteriviridae* family and it can be found in two genotypes (US [type 2] and EU [type 1]). Each genotype comprises of thousands of genetic and antigenic heterogenic strains [147]. PRRSV replicates in cells of the monocyte/macrophage lineage, especially in activated macrophages. Its high variability and its ability for immune evasion make it extremely difficult to design sustainable vaccines, especially under heterologous situations (Wilkinson et al. [130]). Thus, other solutions are searched for to combat the disease, among them the use of genetic resistant pigs.

## Natural genetic disease resistance against PRRS in swine breeds and populations

Halbur et al. [38] provided initial indications of genetic differences in susceptibility/resistance of pigs against PRRS. Duroc pigs showed lower performance combined with an increased severity of lung lesions and antibody titres after infection with PRRSV than Meishan pigs. Clinical abortion rates were found to be associated with IFNγ and influenced by sows’ genetics [67]. A genetic background for differences in performance, severity of lesions, viral titres, infected macrophages and immunological parameters has also been described by Petry et al. [85], Vincent et al. [119, 120], Doeschl-Wilson et al. [26] and Reiner et al. [91], although differences were often small and partially inconsistent over time. Lean lines (Duroc and Hampshire) have been found to be more susceptible than lines selected for higher reproductivity. Ait-Ali et al. [1] reported on favourable macrophages in Landrace pigs and assumed the density and distribution of *CD169* and IL-8 levels to be critical factors. High levels of IL-8 and low levels of IFNγ were also associated with PRRSV resistance by Petry et al. [86].

## PRRS resistance: tracking down the molecular basis by genome-wide genetic association and differential expression studies

These results provided enough evidence for a genetic background of PRRS resistance and remarkable differences in susceptibility between breeds or at least populations. For the next step, pigs differing at most in susceptibility/resistance were used in experiments to take a detailed look at their genetic peculiarities. Three major setups were applied initially: QTL analysis [39] and genome-wide association study (GWAS) were used to identify chromosomal areas and eventually single nucleotide polymorphisms (SNPs) associated with PRRS phenotypes (e.g., degree of viremia, lung lesions and performance after PRRSV infection [13–15], antibody response [105]) and differential expression experiments to detect genes via differences in their expression levels in susceptible and resistant pigs [103, 104]. The most significant results have been achieved by Joan Lunney (USDA), Bob Rowland (Kansas State University) and colleagues, particularly in the context of the PRRS Host Genetics Consortium (PHGC, for a review, see [69]).

Based on up to 60,000 SNP markers together with new statistical tools, more than 30 QTL for resistance against PRRS have been mapped to 11 chromosomes http://www.animalgenome.org/QTLdb/ [13, 15]. As part of the PRRS Host Genetics Consortium, a genome-wide association study based on 190 pigs from a commercial breeding line and the Illumina PorcineSNP60 BeadChip detected associations with viral load and body weight after PRRSV infection. A major QTL region was mapped to chromosome 4 (SSC4), explaining 16 % of genetic variance for virus load with a frequency for the favourable allele of 0.16 and a heritability of 0.30 [13].

One of the most limiting factors in association studies has been the density of gene markers. Next generation sequencing is a recent technological breakthrough that is speeding up the genetics and genomics of a broad range of traits, conferring new opportunities for high-throughput low cost genotyping. Costs for sequencing have dropped by 1:100,000 during the last 15 years. Marker density can be increased by 10^4^ to 10^5^ as compared to conventional SNP-chips which has led to the concept of genotyping by sequencing (for overview see [51]). Such technics may help to raise our understanding of host-PRRSV interaction to a higher end much more complex level, including the complete genomic information of both the host and the virus. Next generation sequencing will have a high impact on the understanding of the virus’ adaption to replication in the host [18, 68].

### GBP5 is an important candidate gene for PRRS resistance

The highest linkage disequilibrium was found for SNP WUR10000125. The interferon-induced guanylate-binding protein 5 gene (*GBP5*) was identified as the most likely candidate in a total of eight consecutive and independent trials [13–15]. This gene was differentially expressed and validated in different pig populations [53] and an intronic SNP (rs340943904) (close to WUR10000125, but not on the 60 k SNP chip) was found to be responsible for introducing a splicing site that truncated the C-terminal 88 amino acids in the recessive A-allele. *GBP5* is involved in immune response to bacterial and viral infection in different species, namely in the inflammatory response and the assembly of the inflammasome in mammals [107], which strongly depends on the C-terminal 67 amino acids which are highly conserved between species [12]. Although the exact role of *GBP5* in PRRSV defence remains to be identified, this SNP is the putative quantitative trait nucleotide (QTN) (i.e., the SNP most likely to be responsible for the QTL on SSC4). In addition, Boddicker et al. [14] only found small effects for resistance to PRRS on SSC1, 5, 7 and X. Further research is needed to show the generality of these findings in other global pig breeds.

A second approach to detect underlying molecular differences in PRRS susceptibility/resistance was performed via microarray-based gene expression analysis, in vivo [2, 6, 9, 11, 36, 42, 46, 75, 103, 104, 137–141, 146] or in vitro [98, 99]. Several immune response pathways were upregulated after infection and several hundreds of differentially expressed genes were detected, but this did not lead to a simple identification of directly responsible genes. One major concern with differential expression (DE) studies is that many differentially expressed genes (A) do not necessarily need to carry the responsible mutation. Instead, their differential expression is achieved via the products of other genes (B) that regulate gene expression by binding to the promoter, the 5′ and 3′ untranslated region or to other regulatory elements of the A genes. These B genes, however, do not necessarily need to be differentially expressed, provided the relevant mutation leads to an amino acid exchange, resulting in altered efficiency of the gene products of genes B at the promoters of genes A. Thus, they may not be detected in DE studies. Thus, the strength of DE studies lies mainly in the detection of the gene networks and pathways involved and in integrating the analysis of genetic and DE data.

## The role of genetic variation in type I interferon genes

Type I interferons are a heterogeneous group of cytokines, important in antiviral response. Genetic variation has been linked to susceptibility to viral diseases, and PRRSV has been found to suppress type I IFN production as a major strategy for evading the immune system [60, 80, 82]. Sang et al. [97] discovered more than 100 polymorphisms in 39 functional genes from the type I interferon family. More than 20 polymorphic mutants have been linked with differing anti-PRRSV activities in vitro [97].

## Genetic variation in autochtonous breeds may contribute genetic resistance against PRRS

Rare breeds, often autochthonous to some regions or countries and poorly adapted to modern pig production, are a valuable source of rare gene variants with sometimes unexpected effects. Rare or even lost SNPs might be (re-)introduced via gene editing methods or by genetic introgression. However, this requires knowledge of these effects and, therefore, the breeds carrying the rare SNPs. One potential example was provided by Li et al. [62] who identified an *Mx1* (myxovirus resistance protein 1) promoter variation, potentially associated with PRRS resistance. *Mx1* exhibits potent anti-RNA viral activity [7, 78] and is involved in early host defence against PRRSV [19, 145]. A second candidate gene, potentially involved in PRRSV resistance, with the valuable allele preferentially restricted to Chinese autochthone breeds, is the ubiquitin-specific protease 18 (*USP18*; [63]).

### The role of microRNA genes

MicroRNAs are small non-coding RNA, involved in post-transcriptional gene regulation [92]. They modify mRNA stability by interaction with its 3′ untranslated region and have been shown to be involved in viral pathogenesis in pigs, e.g. swine influenza virus and pseudorabies virus (He et al. [40]; Anselmo et al. [5]; Loveday et al. [66]). Up to now, no microRNA variability has been described in association with PRRS resistance/susceptibility. However, the porcine microRNAome has been studied in PRRSV-infection and the expression of several microRNAs is altered by PRRSV infection [42, 47, 64]. These results could lead to microRNA-based anti-PRRSV therapies in the future.

## Support from basic virus research: the PRRSV receptors

Most genes and molecules involved in PRRS pathogenesis escape detection via genetic and genomic methods, if they are not variable in sequence or expression, or if this variability is not present in the studied populations. Thus, basic virus research is of high importance in the attempt to resolve the pathogenesis of PRRS and to detect candidate genes for PRRS-resistance.

At least six cellular molecules have been described so far as putative receptors for PRRSV, including *CD163*, the cysteine-rich scavenger receptor (SRCR; [17]), sialoadhesin (*CD169*; siglec-1; [29]), *CD151* [106], heparin sulfate [50], vimentin [52] and *CD209* [45], reviewed by Zhang and Yoo [144].

### CD163

*CD163* is restrictively expressed in cells from the monocyte/macrophage lineage, and significant expression is exclusively found in activated (major) tissue macrophages, together with complement and Fc receptors, other scavenger receptors, and receptors for mediators, adhesion molecules and growth factors [3, 112]. Macrophages not or only newly involved in inflammation and defence do not express *CD163* to any substantial degree [8, 118]. Activation of TLRs (e.g., TLR4) by LPS or other pathogen-associated molecular patterns (PAMPs) increases IL10 [125], one of the strongest upregulators of *CD163* in humans [133]. A second important activator of *CD163* is stress (glucocorticoids) [43, 112].

One major function of *CD163* is in the receptor-mediated endocytosis that delivers extracellular substrates to the endo- and lysosomes of scavenger cells for intracellular metabolism and activation of ligand-specific signal pathways that direct the right answer to the respective substrate [113]. While ligands are delivered to early endosomes, *CD163* recycles to the plasma membrane for new rounds of endocytosis [102]. These events are best recognized regarding the elimination of toxic cell-free haemoglobin from the serum as an important physiological metabolic pathway [56, 102]. Another role of the scavenger receptor seems to be the receptor-mediated internalisation of pathogens, and coincidentally its role as an innate immune sensor for Gram-positive and Gram-negative bacteria, linking bacterial infection with inflammation (e.g., via pro-inflammatory cytokines like TNFα [116]). However, some pathogens have developed mechanisms to evade these physiological processes and use the receptor to enter their host cells, namely African swine fever virus (ASFV; [96]) and PRRSV [17, 83, 114].

### CD163 and PRRSV

*CD163* has been well documented as attachment and internalization receptor in ASFV [96], the PRRSV-related Simian Haemorrhagic Fever Virus (SHFV; [16]) and PRRSV [17]. PRRSV was first identified to enter the cell via a common receptor-dependent endocytosis [55], relying on the clathrin-mediated pathway and a low pH and shifting the virus from the cell surface to early endosomes [79]. The receptor was found to be responsible for the highly specific tropism of the virus [54]. Since then, several attachment factors have been studied extensively as potential PRRSV receptors and *CD163* and *CD169* were identified the most likely candidates involved. However, only *CD163* has been shown capable of conferring PRRSV permissiveness to cell lines unsusceptible to PRRSV, even in the absence of *CD169* (e.g., [17, 83, 113, 114, 116, 123, 124]). It was shown that PRRSV permissivity was conferred by *CD163* independent of the PRRSV genotype involved (1 [EU] or 2 [US]) [17, 61]. Down-regulation of *CD163* (but not *CD169*) in susceptible cells by ADAM17 was able to completely block PRRSV infection [37]. All these data show that *CD163* alone can transfer PRRSV permissiveness to non-responsive cells and establish a productive replication cycle [144]. The role of *CD163* was finally proven in the gene editing experiments of Prather et al. [87] and Whitworth et al. [128], who transferred PRRSV resistance to pigs by deleting *CD163* sequences from the pigs’ genome. However, they had no success when deleting *CD169*.

The central role of *CD163* in PRRSV replication has never been in debate. There was just some discussion about the step where the binding between *CD163* and PRRSV would take place. Van Gorp et al. [113–116] provided evidence for first interactions between PRRSV and *CD163* during virus uncoding in early endosomes. However, the lack of measureable amounts of *CD163* in contact with PRRSV on the cell surface might be due to a fast cycling process of *CD163* between cell surface and endosomes as described by Schaer et al. [102] and Zhang and Yoo [144]. Minor differences between experiments in terms of efficiency of PRRSV replication seem to be more a matter of receptor interaction and membrane lipid environment than of differences between PRRSV genotype, although variability of the pathogen itself also affects the quantitative outcome of PRRSV replication.

The fact that not all cells that express *CD163* can be infected by PRRSV which is important for realisation of PRRSV-specific cell tropism [144] and that PRRSV shows a restricted tropism for subsets of porcine macrophages in vivo might be a question of *CD163* quantity or of interaction with other, maybe until now not identified co-receptors [34]. The expression of *CD163* on macrophages in different microenvironments in vivo, may determine the replication efficiency and subsequent virulence of PRRSV [83].

### CD163 domains

*CD163* consists of nine cysteine-rich tandem repeats, forming the extracellular scavenger receptor, a transmembrane domain and the intracellular cytoplasmic tail. Different from the situation with haemoglobin (domains 2 and 3; [30]), the essential parts of *CD163* in PRRSV entry seems to be related to domain 5, the two proline-serine-threonine (PST)-rich regions and a few others, but not with the complete receptor [113]. The first 4 N-terminal domains and the C-terminal 223 residues (cytoplasmic tail) [59] are not relevant for PRRSV-replication. The transmembrane domain is essential, but not specific [127]. The interacting PRRSV glycoproteins responsible for receptor binding and infection are GP2a, GP3, GP4 and E, [110]. GP4 and GP2a are especially important [21]. Replacing ORFs 2a to 4 with EAV ORFs keeps the virus viable and infectious, but protects macrophages from infection [110].

Glycosylation of GP2a and GP4 by glycans can have different effects on PRRSV replication, depending on the PRRSV genotype [22, 126, 134]. However, transitions are fluent, because of the role of lipids and cholesterol from the lipid rafts of the outer plasma membrane that interact with embedded proteins and receptors [28, 142]. As a putative ion channel protein, the E protein is involved in decreasing pH values as a further part of a successful uncoating process [58].

### Supporting receptors

Sialoadhesin (*CD169*) is a transmembrane glycoprotein, a lectin, restricted to activated tissue macrophages [76, 132] and involved in cell-cell interaction. Expression can be induced in macrophages by IFNα and IFNγ during the inflammatory process [95]. The receptor facilitates pathogen interactions and uptake of sialylated pathogens (e.g., HIV [95] and PRRSV [25, 29, 117]. Especially the amino acids S107 and R116 bind sialic acid of PRRSV GP5 [25, 48, 111]. Sialoadhesin seems to facilitate attachment of PRRSV, eventually together with heparin sulfate, and internalisation, but not replication of the virus [24, 114, 116]. A gene editing experiment that deleted *CD169* found full PRRSV-permissive macrophages and unaltered viremia and antibody production in the pigs [87]. The authors conclude that sialoadhesin is not required for PRRSV infection and that the absence of the *CD169* gene neither prevents PRRS nor alters PRRS pathogenesis.

Heparin sulfate is widely distributed on the surface of most mammalian cells. Heparin sulfate, heparin-like proteins and proteoglycans bind to GP5/M heterodimers and the M complex of PRRSV in a virus-dependent manner [23, 50]. Together with sialoadhesin, heparin sulfate seems to propagate the interaction between PRRSV and its specific receptor(s), but heparin sulfate is not necessarily required for PRRSV entry [23].

*CD151* is involved in numerous cell functions and cell signalling [32]. Silencing the gene made susceptible cells resistant, while overexpression made resistant cells susceptible to PRRSV, making *CD151* a key receptor for PRRSV infection [106]. Blocking *CD151* by microRNA (miR506) prevents the cells from being infected [135]. However, *CD151* is restricted to the erythroid cell lineage and is not expressed on macrophages.

Vimentin and *CD209* are further putative receptors that might be involved in varying efficiency of PRRSV binding and replication [45, 52].

## A gene editing breakthrough in PRRS resistance?

All these results regarding PRRSV receptors finally led to gene editing experiments and the knockout of PRRSV-receptor function in *CD169* [87] and *CD163* [128] in gene-edited pigs. Loss of *CD169* did not affect PRRSV replication, but gene-edited pigs without *CD163*-receptor function were protected from PRRSV. The pigs showed no fever, respiratory or other clinical signs, and no lung pathology, viremia or antibody response after inoculation with a NVSL 97–7895 PRRSV isolate in a controlled study. In addition, no problems occurred during pregnancy and growth of the piglets until challenged with the PRRSV isolate at the age of 3 weeks.

## What is gene editing?

The goal of improving livestock genomes by direct manipulation is old. Its development was accompanied by serious problems in terms of site-specificity (precision), efficiency of the methods used and a lack of acceptance in wider society. Thus, unlike transgenic crops, no transgenic livestock has ever gained commercial approval [57]. All these problems may have been overcome with the introduction of gene editing via CRISPR/CAS9 [27]. The system combines an endonuclease with a specific short guiding (sg) RNA sequence. Like a primer in PCR, this sequence provides accurate specificity, while the linked enzyme can cleave and modify the DNA at exactly the position targeted by the sgRNA sequence. The key-step of this method is the double strand break in DNA and the interaction with cellular DNA repair mechanisms that leads to a high degree of failures (50 %) when joining the ends or even higher, when homology directed repair is induced by the introduction of the desired new sequence [27]. The system can also be used in a multiplex manner to edit different genes in one step. However, comparable to the amplification of incorrect sequences by primer mismatching in PCR, care must be taken not to introduce unintended mutations anywhere in the genome at off-target sites. New methods have been developed to minimise the off-target size problem [71]. Originally, the CRSISPR Cas9 system was part of natural, sequence-specific immunity in bacteria, responsible for the introduction of DNA double-strand breaks into invading plasmids and phages [35]. Taken together, concerns about the precision and efficiency of transgenics have been overcome by this new method in previously inconceivable way. The first genome editing experiment in pigs succeeded to resilience the African Swine Fever receptor by its warthog homologue [65].

## Concerns about gene edition as a tool to generate genetic resistance to combat PRRS

### Gene editing and regulation by authorities

Gene editing can introduce mutations to the genome without adding any footprints associated with the technology. Thus, genome modifications cannot be distinguished from natural mutations [57]. Further, vectors to introduce foreign DNA into transgenic organisms, which might prove hazardous to consumers, are no longer needed. Both factors have led to the enthusiastic acceptance of gene editing by most researchers, the scientific community and the industry. Unlike transgenic organisms, gene-edited plants and animals may not need regulatory oversight [70, 122], provided the human germ line is not involved. Animals and products might not even be classified as genetically modified organisms (GMO). However, as the methodology explodes and a vast number of gene-edited livestock will be produced in the coming years, societal interpretation is currently difficult to predict. However, restrictions are likely.

### Patenting gene-edited PRRS resistance

A second concern is related to upcoming patents. Generally, societies have to decide whether naturally occurring receptors or gene variants with a potential to improve health and welfare should be reserved exclusively for certain companies. The future always brings changes and the ability of populations and species to change is based on their genetic variability. As any individual can carry a maximum of two alleles at any position in the genome, resource populations often lose rare alleles with decreasing population size. These alleles, once lost, cannot be reintroduced by gene editing, as their favourable effects have never been documented. A single breed is not enough to fulfil the different demands of diversified markets worldwide. A chance to become resistant to PRRS needs to be retained for other breeds, lines and populations too.

### Side-effects of CD163-edited knockout pigs

The facts outlined above for *CD163* show that this protein has not evolved solely as a PRRSV receptor, but with a broad spectrum of tasks, including the elimination of pathogens other than PRRSV and the regulation of the immune system. *CD163* awaits the discovery and evaluation of further involvements and mechanisms. Any knockout of *CD163* as a whole or in part needs meticulous investigation of impacted pigs under field conditions, including the effects of other pathogens and adverse conditions. Work is currently in progress and results are expected in future.

### Stability of the genetic resistance in the CD163 knockout pig

Will *CD163* knockout protect against other and upcoming PRRSV strains? One common concern surrounding disease resistance is whether pathogens will be able to adapt to host resistance like they acquire resistance to antibiotics. Acquiring resistance is possible in theory, but the method is unlikely to be similar, because there are no plasmids harbouring information for an arbitrary switch to new tropism. Some examples of single mutations provoked tissue or even species shift under “natural” conditions, although species shifts are very rare events in the evolution of most viruses [33]. A prime example is the Influenza A virus (e.g. [72]). Other examples arise from the *Coronaviridae* (e.g., SARS [31]) and TGE/PRCV [10] viruses.

The specific risk for the development of mutations that could alter cell or even species tropism might be high in PRRSV-infected pig herds. As a RNA virus, PRRSV has high mutation rates and the herd situation generally provides conditions that lead to the crowding of different pathogens or strains. Forsberg et al. [33] conclude that a supposed interspecies transmission for PRRSV took place before 1981. However under the conditions of current pig-PRRSV-interaction - including a high degree of adaptation of the virus to its host, an unmanageable multitude of strains and genotypes and highest burdens within pigs and herds - mutations in the PRRSV genome that might overcome *CD163* could arise within a much shorter period.

The tremendous all-or-nothing-principle of *CD163* on PRRSV replication could provide an unique and widespread solution to the PRRS problem. However, because only one receptor is involved, it runs a strong risk of being overcome by one or few SNPs. Work by Frydas et al. [34] indicates that tropism of PRRSV may change, at least for type 1. The fact that some isolates infected significantly more cells in nasal mucosa than others, suggests the potential existence of additional receptors. Up to now, the *CD163-*knockout experiment was only conducted with type 2 isolates.

On the other hand, differences in oligo- or polygenic pathways that are involved in the immune answer to PRRSV infection are much more complex. This complexity hinders their elucidation and the all-or-nothing-principle of resistance. However, if such natural resistance could be implemented, the odds that PRRSV would overcome these genetic changes would decrease. It is impossible to predict exactly what will happen. Some good examples arise from indigenous (autochthone) breeds, evolved under endemic disease challenge. Such breeds have developed sustainable resistance that makes them superior to others. This aspect further underlines the necessity to preserve genetic and breed diversity in swine.

## Conclusion

The detection and knockout of CD163 as the receptor responsible for PRRSV replication in pigs is a milestone in modern pig production. Complete or even partial elimination of PRRSV replication would lead to a significant improvement in the disastrous situation in infected herds, with significant impact on welfare, production efficiency, performance and consumer protection. However, the complete function of the receptor and its reasonable modification still requires elucidation, and the evaluation of other gene variants involved in immunological pathways is just beginning. Thus, the future will see combined efforts to develop and transfer new knowledge to the herd level. The degree of success in using genetic resistance as an alternative in controlling PRRS will be measured in terms of microbiological and health parameters, but also in terms of availability for pig populations all over the world.
